# Effect of *SYTL3*-*SLC22A3* Variants, Their Haplotypes, and G × E Interactions on Serum Lipid Levels and the Risk of Coronary Artery Disease and Ischaemic Stroke

**DOI:** 10.3389/fcvm.2021.713068

**Published:** 2021-08-12

**Authors:** Peng-Fei Zheng, Rui-Xing Yin, Xiao-Li Cao, Wu-Xian Chen, Jin-Zhen Wu, Feng Huang

**Affiliations:** ^1^Department of Cardiology, Institute of Cardiovascular Diseases, The First Affiliated Hospital, Guangxi Medical University, Nanning, China; ^2^Department of Neurology, The First Affiliated Hospital, Guangxi Medical University, Nanning, China

**Keywords:** coronary artery disease, ischaemic stroke, *SYTL3*, *SLC22A3*, single nucleotide polymorphism, haplotype, lipids

## Abstract

**Background:** The current study aimed to investigate the effects of synaptotagmin-like 3 (*SYTL3*) and solute carrier family 22 member 3 (*SLC22A3*) single nucleotide polymorphisms (SNPs) and gene-environment (G × E) interactions on blood lipid levels as well as the risk of coronary artery disease (CAD) and ischaemic stroke (IS) in the Southern Chinese Han population.

**Methods:** The genetic makeup of 6 *SYTL3-SLC22A3* SNPs in 2269 unrelated participants (controls, 755; CAD, 758 and IS, 756) of Chinese Han ethnicity was detected by the next-generation sequencing techniques.

**Results:** The allele and genotype frequencies of the *SYTL3* rs2129209 and *SLC22A3* rs539298 SNPs were significantly different between the case and control groups. The *SLC22A3* rs539298 SNP was correlated with total cholesterol (TC) levels in controls, the rs539298G allele carriers maintained lower TC levels than the rs539298G allele non-carriers. At the same time, the *SLC22A3* rs539298 SNP interacted with alcohol consumption reduced the risk of CAD and IS. The *SYTL3*-*SLC22A3* A-C-A-A-A-A, G-T-C-G-C-A and A-T-A-A-C-A haplotypes increased and the A-C-A-A-C-G haplotype reduced the risk of CAD, whereas the *SYTL3*-*SLC22A3* A-C-A-A-A-A, G-T-C-G-A-G and A-T-A-A-C-A haplotypes increased and the A-C-A-A-A-G and A-C-A-A-C-G haplotypes reduced the risk of IS. In addition, several SNPs interacted with alcohol consumption, body mass index ≥ 24 kg/m^2^ and cigarette smoking to affect serum lipid parameters such as triglyceride, high-density lipoprotein cholesterol, TC, and apolipoprotein A1 levels.

**Conclusions:** Several *SYTL3*-*SLC22A3* variants, especially the rs539298 SNP, several haplotypes, and G × E interactions, were related to blood lipid parameters and the risk of CAD and IS in the Southern Chinese Han population.

## Introduction

Ischaemic cardiovascular and cerebrovascular diseases, including coronary artery disease (CAD) and ischaemic stroke (IS), are the primary contributors to global disability and death despite the vast therapeutic and diagnostic methods innovated in the last 10 years. Several recent studies have shown that as a multifactorial and complex disorder, CAD or IS occurs due to numerous factors, such as unhealthy lifestyles, alterations of serum lipid levels, genomic background and environmental factors and interactions among these factors (1–3). As the common pathological basis of CAD (4) and IS (5), atherosclerosis is the result of chronic inflammation (6) and abnormal lipid metabolism, such as increased levels of triglyceride (TG) (7), apolipoprotein (Apo) B (8), low-density lipoprotein cholesterol (LDL-C) (9), and total cholesterol (TC) (10), along with reduced levels of ApoA1 (8) and high-density lipoprotein cholesterol (HDL-C) (11) in serum.

Many genes and genetic loci associated with CAD (12) or IS (13) have been identified and reported in previous genome-wide association studies (GWASes). Similarly, a large number of genes or loci associated with lipid metabolism have also been associated with CAD and/or IS (1–3). Currently, several compelling genes closely associated with blood lipid parameters, including synaptotagmin-like 3 (*SYTL3*) and solute carrier family 22-member 3 (*SLC22A3*), have been identified in the European population by GWASes (14). The *SYTL3* (also named *SLP3*, gene ID: 94120, OMIM: 608441, HGNC:15587) is located on chromosome 6q25.3 (exon count: 22), and its encoded protein plays a key role in vesicular trafficking. Several compelling studies have suggested that the transport of lipids and proteins between eukaryotic cells via secretion and endocytosis is primarily facilitated by vesicular trafficking (15, 16). Muse et al. (17) reported that the *SYTL3* was associated with the incidence of acute myocardial infarction (AMI). However, its mechanism remains unclear.

The *SLC22A3* (also known as *OCT3*; *EMTH*; *EMT*, gene ID: 6581, OMIM: 604842, HGNC:10967) is located on chromosome 6q25.3 (exon count: 15), and it encodes an organic cation transporter 3 protein, which is a member of the peripheral membrane protein family and plays a key role in biogenic histamine deactivation and synthesis (18, 19). As an effective proinflammatory mediator, histamine can enhance the deposition of LDL-C in vascular endothelial cells by inducing inflammatory mediators such as adhesion molecules, cytokines, chemokines and others. Inflammatory mediators further increase the permeability of vascular endothelial cells, so histamine can create a favorable environment for the formation of atherosclerotic plaques (20, 21). Previous studies showed that the *SLC22A3* silencing could effectively inhibit the synthesis of histamine and proinflammatory mediators (*MCP-1, IL-8* and *IL-6*) and reduce the infiltration of monocytes and the adhesion between leukocytes and the endothelium (22). The *SLC22A3* expression is widespread, and it can be found in skeletal muscle, heart, brain, and placenta. The *SLC22A3* is highly expressed in the human heart, with the strongest *SLC22A3* immunoreactivity found in vascular endothelial cells (23, 24). A series of studies have confirmed that the *SLC22A3* rs1810126, rs2048327 and rs3088442 SNPs reduce the risk of CAD by downregulating *SLC22A3* transcription and protein levels (22, 25). Chen et al. (26) found that the *SLC22A3* could be regarded as a gene vector for the association between lipid metabolism and CAD. Nevertheless, the association between the *SYTL3* rs9364496, rs6455600, rs2129209, rs9456350, *SLC22A3* rs446809 and rs539298 SNPs and the risk of CAD and IS is still unclear and not reported in the Chinese Han population. Thus, this research aimed to understand the relationship between the six selected SNPs and the risk of CAD and IS in the Southern Chinese Han population.

## Methods

### Subjects

A case-control study was conducted among the Chinese Han participants in Guangxi Zhuang Autonomous Region, a province in Southern China. A total of 2,269 samples were collected from the First Affiliated Hospital of Guangxi Medical University, including controls, 755; CAD patients, 758; and IS cases, 756. CAD was defined as significant coronary artery stenosis (≥ 50%) in at least one of the three major coronary arteries or their major branches (branch diameter ≥ 2 mm) (27). All patients suffering from IS received detailed and rigorous neurological examination as well as brain magnetic resonance imaging (MRI) scans. The diagnostic criteria for IS were based on the International Classification of Diseases (9th Revision). Subjects with a history of neoplastic, autoimmune, type 1 diabetes mellitus, haematologic, thyroid, renal or liver diseases were excluded. Patients suffering from IS had no history of CAD, and patients suffering from CAD also had no history of IS.

After adjusting for sex and age, all of the healthy control participants were randomly recruited from the Physical Examination Center of the First Affiliated Hospital of Guangxi Medical University during the same period. All participants were healthy, and none of them had a history of myocardial infarction (MI), type 2 diabetes mellitus (T2DM), CAD or IS as judged by a critical clinical examination and medical history collection. All subjects in the current research signed written informed consent forms. The research proposal was approved by the Ethics Committee of the First Affiliated Hospital, Guangxi Medical University (No: Lunshen-2014-KY-Guoji-001; Mar. 7, 2014).

### Biochemical Index Detection

After fasting for at least 12 h, a sample of 5 ml of peripheral venous blood was drawn from each participant. A portion of the blood sample (2 ml) was used to measure serum lipid levels, and another portion of the blood sample (3 ml) was used to extract genomic DNA. The methods for measuring serum ApoB, LDL-C, ApoA1, TC, HDL-C, and TG were described in detail in our previous study (28). All determinations were performed using an autoanalyser (Type 7170A; Hitachi Ltd., Tokyo, Japan) in the Clinical Science Experiment Center of the First Affiliated Hospital, Guangxi Medical University (29).

### Diagnostic Criteria

The definition for normal values of serum ApoB (0.80–1.05 g/L), TG (0.56–1.70 mmol/L), ApoA1 (1.20–1.60 g/L), LDL-C (2.70–3.10 mmol/L), TC (3.10–5.17 mmol/L), HDL-C (1.16–1.42 mmol/L), and the ApoA1/ApoB ratio (1.00–2.50) was based on our previous study (30). The diagnostic criteria for hyperlipidaemia (31), hypertension (32, 33), obesity, overweight, and normal weight (34, 35) were described in detail in previous studies (31–35). Participants with a previous diagnosis of diabetes or a fasting plasma glucose ≥ 7.0 mmol/L or 2 h postprandial plasma glucose ≥ 11.1 mmol/L were defined as diabetic patients (36).

### SNP Selection and Genotyping

Six SNPs located on *SYTL3* and *SLC22A3* were chosen based on predetermined criteria: (1) The *SYTL3*-*SLC22A3* cluster was selected from previous GWASes associated with blood lipid levels. (2) Tagging SNPs were identified *via* Haploview (Broad Institute of MIT and Harvard, USA, version 4.2), and potential lipid metabolism-associated functional SNPs were predicted using the latest version of the 1000 Genome Project Database. (3) More complete details of the selected SNPs were collected from NCBI dbSNP Build 132. (4) Regarding SNP selection, we also referenced a previous study by Ober et al. (14), and the minor allele frequency (MAF) of all SNPs was more than 1% and associated with blood lipid profiles in a previous study. (5) Six SNPs of *SYTL3* (rs9364496, rs6455600, rs2129209 and rs9456350) and *SLC22A3* (rs446809 and rs539298) were selected using the block-based method, which involves marking the association of linkage disequilibrium (LD) amongst the chosen SNPs (*r*^2^ > 0.8). White blood cells were used for genomic DNA extraction with phenol-chloroform (37). All obtained DNA samples were numbered and maintained at −20°C until further studies were carried out. Next-generation sequencing technology (NGS) was used to analyse the genotypes of the 6 selected SNPs at the Center for Human Genetics Research, Shanghai Genesky Bio-Tech Co. Ltd., China (38). Supplementary Table 1 depicts the relevant primer sequences. Detailed steps for multiplex PCR and high throughput sequencing are also shown in Supplementary Material.

### Statistical Analyses

SPSS (Version 22.0) software was used to analyse all data collected from the current research. An independent sample *t*-test was used to evaluate the continuous data (mean ± SD) that were normally distributed between control and patient groups. TG levels that were not normally distributed were expressed using median and quartile ranges and were evaluated using the Wilcoxon-Mann-Whitney test. Data such as the genotype distribution, sex ratio, and the number of smokers and drinkers were analyzed by the chi-square test. The standard goodness-of-fit test was used to test the Hardy-Weinberg equilibrium (HWE). The correlation between genotypes and blood lipid levels was tested by the covariance analysis (ANCOVA). Any variants related to the blood lipid parameter at a value of *P* ≤ 0.008 were considered statistically significant after Bonferroni correction. Age, alcohol consumption, sex, cigarette smoking and body mass index (BMI) were adjusted for the statistical analysis. Unconditional logistic regression analysis was used to calculate the 95% confidence interval (CI) and the odds ratio (OR). The interactions of the six selected SNPs with cigarette smoking, sex, age, alcohol consumption and BMI ≥ 24 kg/m^2^ on blood lipid levels and the risk of CAD and IS were performed by using a factorial regression analysis after controlling for potential confounders. A *P* ≤ 0.00125 was considered statistically significant after Bonferroni correction. Haploview (Broad Institute of MIT and Harvard, USA, version 4.2) software was used to calculate the haplotype frequencies and pair wise LD among the six detected SNPs. The heatmap of the inter-locus models was measured by using R software (version 4.1.0).

## Results

### Common and Biochemical Characteristics

As presented in Table 1, there were no differences in the proportion of smokers, diastolic blood pressure, age, the sex ratio, or serum ApoB and LDL-C levels between the controls and cases. The glucose, pulse pressure, systolic blood pressure, BMI, serum TG levels were significantly lower, and the serum TC, ApoA1, and HDL-C levels; the proportion of drinkers; and the ApoA1/ApoB ratio were significantly higher in controls than in CAD and IS patients.

**Table 1:** Comparison of demographic, lifestyle characteristics, and serum lipid levels of the participants.

**Characteristic**	**Control**	**Case**			
	**(*n* = 755)**	**CAD (*n* = 758)**	**IS (*n* = 756)**	***P*_**Control vs. CAD**_**	***P*_**Control vs. IS**_**
Male/female	575/180	553/205	602/154	0.153	0.104
Age (years)	61.86 ± 9.96	62.63 ± 9.46	62.36 ± 10.97	0.270	0.354
BMI (kg/m^2^)	22.57 ± 2.94	23.60 ± 2.96	23.61 ± 3.48	0.000	0.000
Smoking, *n* %	322 (42.6)	346 (45.6)	354 (46.8)	0.240	0.103
Alcohol, *n* %)	349 (46.2)	205 (27.0)	212 (28.0)	0.000	0.000
SBP (mmHg)	128.69 ± 19.29	134.27 ± 23.05	147.86 ± 21.79	0.000	0.000
DBP (mmHg)	80.84 ± 11.56	79.89 ± 13.85	82.00 ± 12.60	0.144	0.064
PP (mmHg)	47.84 ± 14.48	54.39 ± 18.27	65.86 ± 18.07	0.000	0.000
Glu (mmol/L)	6.00 ± 1.57	6.32 ± 1.54	6.19 ± 1.43	0.000	0.014
TC (mmol/L)	4.89 ± 0.96	4.49 ± 0.99	4.54 ± 1.03	0.000	0.002
TG (mmol/L)	1.03 (0.73)	1.30 (0.93)	1.41 (0.97)	0.000	0.000
HDL-C (mmol/L)	1.91 ± 0.51	1.13 ± 0.34	1.22 ± 0.43	0.000	0.000
LDL-C (mmol/L)	2.74 ± 0.78	2.71 ± 0.97	2.77 ± 0.91	0.480	0.577
ApoA1 (g/L)	1.41 ± 0.27	1.03 ± 0.36	1.02 ± 0.22	0.000	0.000
ApoB (g/L)	0.89 ± 0.21	0.89 ± 0.26	0.91 ± 0.24	0.739	0.078
ApoA1/ApoB	1.68 ± 0.64	1.27 ± 0.79	1.21 ± 0.48	0.000	0.000

*SBP, systolic blood pressure; DBP, diastolic blood pressure; PP, pulse pressure; Glu, glucose; HDL-C, high-density lipoprotein cholesterol; LDL-C, low-density lipoprotein cholesterol; Apo, apolipoprotein; TC, total cholesterol; TG, triglyceride. The value of triglyceride was presented as median (interquartile range), the difference between the control and CAD/IS groups was determined by the Wilcoxon-Mann-Whitney test*.

### Genotypic and Allelic Frequencies

As shown in Table 2, all detected SNPs in the CAD, IS and control groups conformed to HWE (*P* > 0.05). The frequencies of the rs2129209AC/CC genotypes and the rs2129209C allele were lower in CAD (AC/CC, 43.7%; C, 25.1%) and IS (AC/CC, 44.1%; C, 24.6%) patients than in control participants (AC/CC, 50.3%; C, 28.6%, *P* < 0.05-0.01 for all). The frequencies of the rs539298AG/GG genotypes and rs539298G allele were also lower in CAD (AG/GG, 42.1%; G, 23.7%) and IS (AG/GG, 40.2%; G, 22.7%) patients than in control subjects (AG/GG, 49.3%; G, 28.3%, *P* < 0.05-0.001 for all). The specific and detailed genotype distribution of the six selected SNPs in the control, CAD, and IS groups is shown in Supplementary Material.

**Table 2:** Genotype and allele frequencies of the 6 *SYTL3*- *SLCC2A3* SNPs in cases and controls [*n*(%)].

**Genotype**	**Control (*n* 755)**	**CAD (*n* = 758)**	**IS (*n* = 756)**	**Allele**	**Control (*n* = 1,510)**	**CAD (*n* = 1,516)**	**IS (*n* = 1,512)**
**rs9364496**							
AA	481 (63.7)	448 (59.1)	486 (64.3)				
AG	237 (31.4)	276 (36.4)	234 (30.9)	A	1199 (79.4)	1172 (77.3)	1206 (79.8)
GG	37 (4.9)	34 (4.5)	36 (4.8)	G	311 (21.6)	344 (23.7)	306 (20.2)
*x* ^2^		4.258	0.058			1.958	0.060
*P*		0.119	0.971			0.162	0.807
*P*_HWE_	0.268	0.298	0.257				
**rs6455600**							
CC	365 (48.3)	364 (48.0)	356 (47.1)				
CT	332 (44.0)	332 (43.8)	327 (43.2)	C	1062 (70.3)	1060 (69.9)	1039 (68.7)
TT	58 (7.7)	62 (8.2)	73 (9.7)	T	448 (29.7)	456 (30.1)	473 (31.3)
*x* ^2^		0.129	1.867			0.061	0.929
*P*		0.938	0.393			0.805	0.335
*P*_HWE_	0.140	0.256	0.868				
**rs2129209**							
AA	375 (49.7)	427 (56.3)	423 (56.0)				
AC	328 (43.4)	282 (37.2)	294 (38.9)	A	1078 (71.4)	1136 (74.9)	1140 (75.4)
CC	52 (6.9)	49 (6.5)	39 (5.2)	C	432 (28.6)	380 (25.1)	372 (24.6)
*x* ^2^		6.924	6.602			4.838	21.577
*P*		0.031	0.037			0.028	0.001
*P*_HWE_	0.081	0.791	0.185				
**rs9456350**							
AA	572 (75.8)	595 (78.5)	568 (75.1)				
AG	173 (22.9)	153 (20.2)	178 (23.5)	A	1317 (87.2)	1343 (88.6)	1314 (86.9)
GG	10 (1.3)	10 (1.3)	10 (1.3)	G	193 (12.8)	173 (10.4)	198 (12.1)
*x* ^2^		1.674	0.085			1.335	0.066
*P*		0.433	0.959			0.248	0.797
*P*_HWE_	0.446	0.963	0.343				
**rs446809**							
CC	378 (50.1)	370 (48.8)	360 (47.6)				
CA	315 (41.7)	318 (42.0)	331 (43.8)	C	1071 (70.9)	1058 (69.8)	1051 (69.5)
AA	62 (8.2)	70 (9.2)	65 (8.6)	A	439 (28.1)	458 (29.2)	461 (30.5)
*x* ^2^		0.579	0.906			1.728	1.454
*P*		0.749	0.636			0.189	0.228
*P*_HWE_	0.749	0.888	0.365				
**rs539298**							
AA	383 (50.7)	439 (57.9)	452 (59.8)				
AG	317 (42.0)	279 (36.8)	265 (35.0)	A	1083 (71.7)	1157 (76.3)	1169 (77.3)
GG	55 (7.3)	40 (5.3)	39 (5.2)	G	427 (28.3)	359 (23.7)	343 (22.7)
*x* ^2^		8.600	13.242			8.316	12.447
*P*		0.014	0.001			0.004	0.000
*P*_HWE_	0.335	0.614	0.984				

*CAD, coronary artery disease; IS, ischemic stroke*.

### Genotypes and the Risk of Diseases

As described in Table 3, only the rs539298 SNP decreased the risk of CAD and IS (*P* < 0.008 was considered statistically significant after Bonferroni correction). The dominant model (AG/GG *vs*. AA) for CAD group showed OR = 0.74, 95% CI = 0.60–0.91, *P* = 0.0053; and the log-additive model (A vs. G) illustrated OR = 0.76, 95% CI = 0.64–0.91, *P* = 0.002. The dominant model (AG/GG *vs*. AA) for IS group displayed OR = 0.67, 95% CI = 0.54–0.83, *P* = 2E-04; and the log-additive model (A vs. G) revealed OR = 0.74, 95% CI = 0.62–0.88, *P* = 6E-04.

**Table 3:** Genotypes of the six *SYTL3*-*SLC22A3* SNPs and the risk of CAD and IS.

**SNP/model**	**Ref. genotype**	**Effect genotype**	**CAD (OR 95% CI)**	***P*_**CAD**_**	**IS (OR 95% CI)**	***P*_**IS**_**
**rs9364496**						
Codominant	A/A	A/G	1.26 (1.01–1.59)	0.12	0.99 (0.79–1.25)	0.99
		G/G	1.01 (0.61–1.67)		0.96 (0.59–1.58)	
Dominant	A/A	A/G+G/G	1.23 (0.99–1.53)	0.064	0.99 (0.79–1.23)	0.93
Recessive	A/A+A/G	G/G	0.92 (0.56–1.52)	0.76	0.96 (0.59–1.58)	0.89
Overdominant	A/A+G/G	A/G	1.26 (1.01–1.58)	0.041	1.00 (0.79–1.25)	0.98
Log-additive			1.14 (0.95–1.37)	0.15	0.99 (0.82–1.18)	0.9
**rs6455600**						
Codominant	C/C	C/T	1.09 (0.87–1.36)	0.74	1.01 (0.81–1.27)	0.61
		T/T	1.10 (0.73–1.64)		1.22 (0.82–1.81)	
Dominant	C/C	C/T+T/T	1.09 (0.88–1.35)	0.44	1.05 (0.85–1.29)	0.68
Recessive	C/C+C/T	T/T	1.06 (0.71–1.57)	0.78	1.21 (0.83–1.77)	0.32
Overdominant	C/C+T/T	C/T	1.07 (0.86–1.33)	0.53	0.98 (0.79–1.22)	0.89
Log-additive			1.06 (0.90–1.26)	0.47	1.07 (0.90–1.26)	0.45
**rs2129209**						
Codominant	A/A	A/C	0.82 (0.65–1.02)	0.18	0.81 (0.65–1.02)	0.063
		C/C	0.82 (0.53–1.27)		0.65 (0.41–1.03)	
Dominant	A/A	A/C+C/C	0.82 (0.66–1.01)	0.062	0.79 (0.64–0.98)	0.031
Recessive	A/A+A/C	C/C	0.90 (0.59–1.37)	0.62	0.71 (0.46–1.12)	0.14
Overdominant	A/A+C/C	A/C	0.83 (0.67–1.04)	0.1	0.85 (0.69–1.05)	0.14
Log-additive			0.86 (0.73–1.02)	0.086	0.81 (0.68–0.97)	0.019
**rs9456350**						
Codominant	A/A	A/G	0.89 (0.69–1.16)	0.67	0.99 (0.77–1.28)	0.96
		G/G	0.85 (0.34–2.12)		1.13 (0.46–2.81)	
Dominant	A/A	A/G+G/G	0.89 (0.69–1.15)	0.37	1.00 (0.78–1.28)	0.99
Recessive	A/A+A/G	G/G	0.87 (0.35–2.17)	0.76	1.13 (0.46–2.81)	0.79
Overdominant	A/A+G/G	A/G	0.90 (0.69–1.16)	0.41	0.99 (0.77–1.28)	0.95
Log-additive			0.90 (0.72–1.13)	0.37	1.01 (0.80–1.27)	0.94
**rs446809**						
Codominant	C/C	C/A	1.15 (0.92–1.45)	0.064	1.20 (0.96–1.51)	0.044
		A/A	1.58 (1.06–2.36)		1.65 (1.08–2.53)	
Dominant	C/C	C/A+A/A	1.22 (0.98–1.52)	0.08	1.25 (1.00–1.57)	0.046
Recessive	C/C+C/A	A/A	1.47 (1.01–2.15)	0.046	1.48 (0.99–2.22)	0.054
Overdominant	C/C+A/A	C/A	1.07 (0.86–1.33)	0.55	1.11 (0.89–1.37)	0.37
Log-additive			1.22 (1.03–1.44)	0.024	1.25 (1.05–1.49)	0.014
**rs539298**						
Codominant	A/A	A/G	0.77 (0.62–0.96)	0.0082	0.67 (0.53–0.84)	0.0511
		G/G	0.56 (0.36–0.88)		0.66 (0.42–1.04)	
Dominant	A/A	A/G+G/G	0.74 (0.60–0.91)	0.0053	0.67 (0.54–0.83)	2E-04
Recessive	A/A+A/G	G/G	0.63 (0.40–0.98)	0.038	0.79 (0.51–1.22)	0.28
Overdominant	A/A+G/G	A/G	0.82 (0.66–1.02)	0.07	0.70 (0.56–0.87)	0.0012
Log-additive			0.76 (0.64–0.91)	0.002	0.74 (0.62–0.88)	6E-04

*SNP, single nucleotide polymorphism; CAD, coronary artery disease; IS, ischemic stroke. Adjusted for sex, age, smoking, drinking, BMI, diabetes, hypertension*.

### Interaction Between the rs539298 SNP and Environmental Factors on the Risk of Diseases

**Figure 1:**
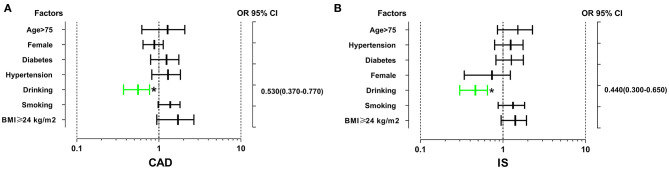
The interactions of the *SLC22A3* rs539298 SNP and diabetes, drinking, hypertension, smoking, age, sex, and BMI on the risk of CAD and IS. The rs539298AG/GG genotypes interacted with alcohol consumption to decrease the risk of CAD **(A)** (OR = 0.53, 95% CI = 0.37–0.77) and IS (**B)** (OR = 0.44, 95% CI = 0.30–0.65). *P < 0.01.

As shown in Figure 1, the interaction between the rs539298AG/GG genotypes and alcohol consumption reduced the risk of CAD (OR = 0.53, 95% CI = 0.37–0.77, *P* < 0.01) and IS (OR = 0.44, 95% CI = 0.30–0.65, *P* < 0.01).

### Haplotypes and the Risk of Diseases

**Figure 2:**
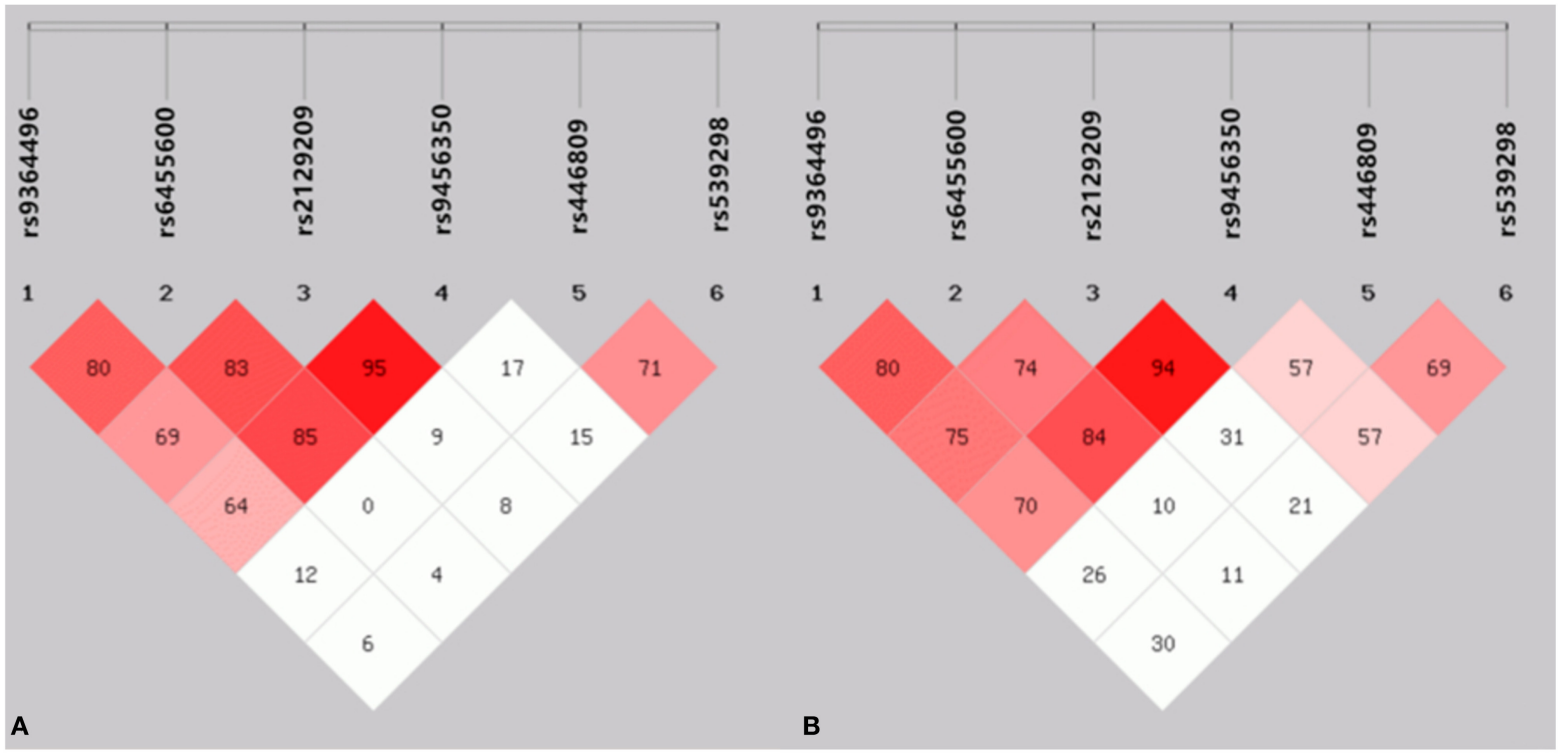
The linkage disequilibrium (LD) represents pair wise *D*' × 100 in the CAD **(A)** and IS **(B)** groups.

As presented in Figure 2, strong LD was found among the *SYTL3* rs9364496, rs6455600, rs2129209 and rs9456350 SNPs as well as the *SLC22A3* rs446809 and rs539298 SNPs in the CAD (Figure 2A) and IS (Figure 2B) groups (*D'* = 0.64–0.95).

Haplotype analysis among the six selected SNPs showed that the main haplotype was the *SYTL3*-*SLC22A3* A-C-A-A-C-A (Table 4). The *SYTL3*-*SLC22A3* A-C-A-A-A-A (adjusted OR = 2.19, 95% CI = 1.49–3.21, *P* = 1E-04), G-T-C-G-C-A (adjusted OR = 1.82, 95% CI = 1.07–3.10, *P* = 0.026) and A-T-A-A-C-A (adjusted OR = 2.04, 95% CI = 1.14–3.66, *P* = 0.016) haplotypes increased the risk of CAD, while the *SYTL3*-*SLC22A3* A-C-A-A-C-G (adjusted OR = 0.52, 95% CI = 0.34–0.78, *P* = 0.0016) haplotype reduced the risk of CAD. Meanwhile, the *SYTL3*-*SLC22A3* A-C-A-A-A-A (adjusted OR = 1.98, 95% CI = 1.39–2.84, *P* = 2E-04), G-T-C-G-A-G (adjusted OR = 2.79, 95% CI = 1.87–4.15, *P* < 0.0001) and A-T-A-A-C-A (adjusted OR = 3.24, 95% CI = 1.92–5.48, *P* < 0.0001) haplotypes increased the risk of IS, whereas the *SYTL3*-*SLC22A3* A-C-A-A-A-G (adjusted OR = 0.63, 95% CI = 0.42–0.95, *P* = 0.027) and A-C-A-A-C-G (adjusted OR = 0.59, 95% CI = 0.40–0.88, *P* = 0.0099) haplotypes reduced the risk of IS.

**Table 4:** Haplotype frequencies of the six *SYTL3*-*SLC22A3* SNPs and the risk of CAD and IS.

**Haplotype**	**Control frequency**		**CAD**			**IS**	
		**Frequency**	**OR (95% CI)**	***P***	**Frequency**	**OR (95% CI)**	***P***
A-C-A-A-C-A	0.4458	0.4446	1.00	—	0.4813	1.00	—
A-C-A-A-A-G	0.1052	0.1242	1.32 (0.94–1.84)	0.11	0.0528	0.63 (0.42–0.95)	0.027
A-C-A-A-A-A	0.0362	0.0645	2.19 (1.49–3.21)	1E-04	0.0641	1.98 (1.39–2.84)	2E-04
G-T-C-A-C-A	0.0398	0.0573	1.18 (0.78–1.79)	0.43	0.0357	0.94 (0.58–1.54)	0.820
G-T-C-G-A-G	0.0448	0.0403	0.97 (0.61–1.56)	0.91	0.1175	2.79 (1.87–4.15)	<0.0001
A-C-A-A-C-G	0.0486	0.0236	0.52 (0.34–0.78)	0.0016	0.0291	0.59 (0.40–0.88)	0.0099
G-T-C-G-C-A	0.0264	0.0472	1.82 (1.07–3.10)	0.026	0	–	–
A-T-C-A-C-A	0.0330	0.0366	1.13 (0.69–1.85)	0.64	0.0341	0.75 (0.46–1.23)	0.25
A-T-A-A-C-A	0.0195	0.0359	2.04 (1.14–3.66)	0.016	0.0753	3.24 (1.92–5.48)	<0.0001

*CAD, coronary artery disease; IS, ischemic stroke. The haplotypes consist of six alleles in the order of rs9364496, rs6455600, rs2129209, rs9456350, rs446809, and rs539298 SNPs. Adjusted for sex, age, smoking, drinking, BMI, diabetes, hypertension*.

### Interactions Between the Haplotypes and Environmental Factors on the Risk of Diseases

**Figure 3:**
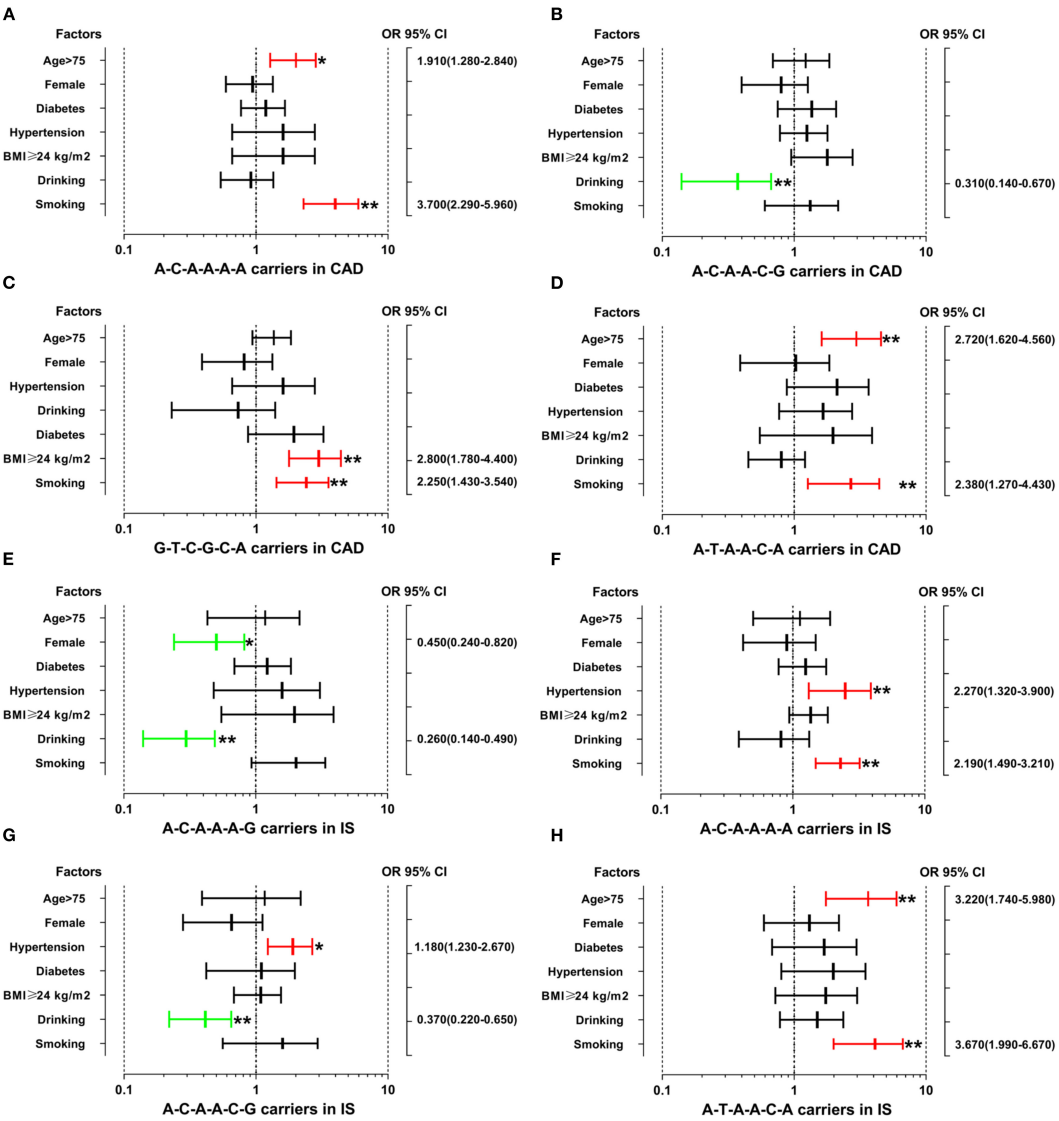
The interactions between the *SYTL3*-*SLC22A3* haplotypes and the risk of CAD and IS. CAD, coronary artery disease; IS, ischaemic stroke. **(A)** The *SYTL3*-*SLC22A3* A-C-A-A-A-A haplotype interacted with age > 75 and smoking to increase the risk of CAD. **(B)** The interaction between the *SYTL3*-*SLC22A3* A-C-A-A-C-G haplotype and alcohol consumption decreased the risk of CAD. **(C)** The interactions between *SYTL3*-*SLC22A3* G-T-C-G-C-A and cigarette smoking and BMI ≥ 24 kg/m^2^ increased the risk of CAD. **(D)** The *SYTL3*-*SLC22A3* A-T-A-A-C-A haplotype interacted with age > 75 and smoking to increase the risk of CAD. **(E)** The interactions between the *SYTL3*-*SLC22A3* A-C-A-A-A-G haplotype and female and alcohol consumption decreased the risk of IS. **(F)** The *SYTL3*-*SLC22A3* A-C-A-A-A-A haplotype interacted with hypertension and smoking to increase the risk of IS. **(G)** The interactions between the *SYTL3*-*SLC22A3* A-C-A-A-C-G haplotype and hypertension increased the risk of IS, and alcohol consumption decreased the risk of IS. **(H)** The interactions between the *SYTL3*-*SLC22A3* A-T-A-A-C-A haplotype and age > 75 and smoking increased the risk of IS. *P < 0.05 and **P < 0.01.

The interactions between the *SYTL3*-*SLC22A3* haplotypes and several environmental factors on the risk of CAD and IS are shown in Figure 3. The interactions of the *SYTL3*-*SLC22A3* A-C-A-A-A-A-smoking (adjusted OR = 3.70, 95% CI = 2.29–5.96, *P* < 0.01), *SYTL3*-*SLC22A3* A-C-A-A-A-A-age > 75 (adjusted OR = 1.91, 95% CI = 1.28–2.84, *P* < 0.05), *SYTL3*-*SLC22A3* G-T-C-G-C-A-smoking (adjusted OR = 2.25, 95% CI = 1.43–3.54, *P* < 0.01), *SYTL3*-*SLC22A3* G-T-C-G-C-A-BMI ≥ 24 kg/m^2^ (adjusted OR = 2.80, 95% CI = 1.78–4.40, *P* < 0.01), *SYTL3*-*SLC22A3* A-T-A-A-C-A-age > 75 (adjusted OR = 2.72, 95% CI = 1.62–4.56, *P* < 0.01), and *SYTL3*-*SLC22A3* A-T-A-A-C-A-smoking (adjusted OR = 2.38, 95% CI = 1.27–4.43, *P* < 0.01) increased the risk of CAD, whereas the interaction of the *SYTL3*-*SLC22A3* A-C-A-A-C-G-drinking (adjusted OR = 0.31, 95% CI = 0.14–0.67, *P* < 0.01) decreased the risk of CAD.

The interactions of the *SYTL3*-*SLC22A3* A-C-A-A-A-A-hypertension (adjusted OR = 2.27, 95% CI = 1.32–3.90, *P* < 0.01), *SYTL3*-*SLC22A3* A-C-A-A-A-A-smoking (adjusted OR = 2.19, 95% CI = 1.49–3.21, *P* < 0.01), *SYTL3*-*SLC22A3* A-C-A-A-C-G-hypertension (adjusted OR = 1.18, 95% CI = 1.23–2.67, *P* < 0.05), *SYTL3*-*SLC22A3* A-T-A-A-C-A-age > 75 (adjusted OR = 3.22, 95% CI = 1.74–5.98, *P* < 0.01), *SYTL3*-*SLC22A3* A-T-A-A-C-A-smoking (adjusted OR = 3.67, 95% CI = 1.99–6.67, *P* < 0.01) increased the risk of IS, whereas the interactions of the *SYTL3*-*SLC22A3* A-C-A-A-A-G-female (adjusted OR = 0.45, 95% CI = 0.24–0.82, *P* < 0.05), *SYTL3*-*SLC22A3* A-C-A-A-A-G-drinking (adjusted OR = 0.26, 95% CI = 0.14–0.49, *P* < 0.01), and *SYTL3*-*SLC22A3* A-C-A-A-C-G-drinking (adjusted OR = 0.37, 95% CI = 0.22–0.65, *P* < 0.01) decreased the risk of IS. However, there was no significant interaction between the *SYTL3*-*SLC22A3* G-T-C-G-A-G haplotype and several environmental factors including gender, age, diabetes, hypertension, BMI, smoking and drinking.

### Relationship Between Genotypes and Serum Lipid Parameters

**Figure 4:**
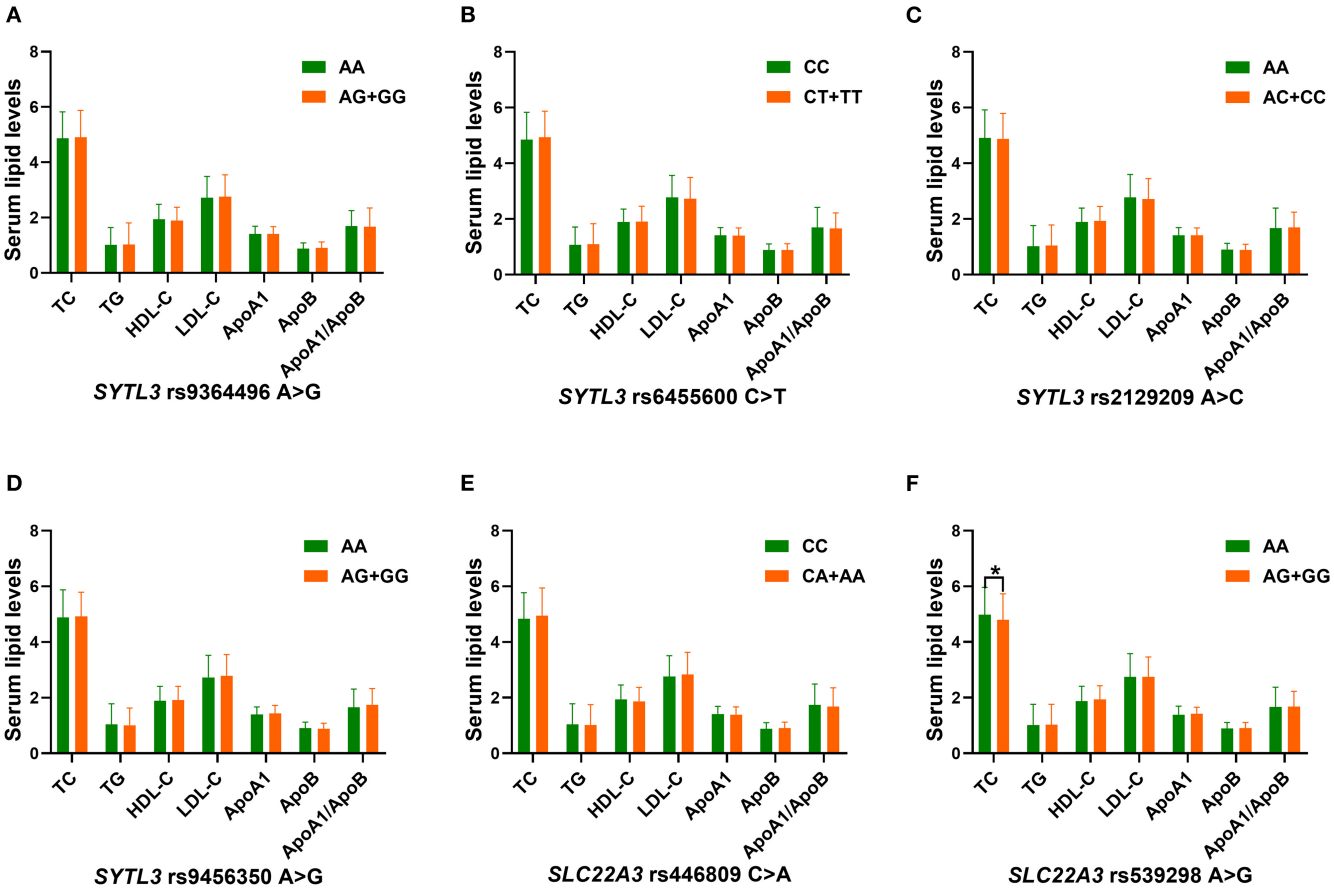
The association between the genotypes of six *SYTL3*-*SLC22A3* SNPs and blood lipid levels in the control group. HDL-C, high-density lipoprotein cholesterol; TC, total cholesterol; ApoA1, apolipoprotein A1; TG, triglyceride; ApoB, apolipoprotein B; LDL-C, low-density lipoprotein cholesterol; ApoA1/ApoB, the ratio of apolipoprotein A1 to apolipoprotein B. A *P < 0.008 (after adjusting for 6 independent tests by the Bonferroni correction) was considered statistically significant. **(A)**, rs9364496; **(B)**, rs6455600; **(C)**, rs2129209; **(D)**, rs9456350; **(E)**, rs446809; and **(F)**, rs539298 SNPs.

The correlation between the *SYTL3*-*SLC22A3* SNPs and serum lipid parameters in the control group is shown in Figure 4. There were significant differences in TC levels between the rs539298AA and rs539298AG/GG genotypes (*P* < 0.008 was considered statistically significant after Bonferroni correction), the subjects with rs539298AG/GG genotypes had lower serum TC levels than those with rs539298AA genotype. However, there was no significant correlation between the remaining 5 SNPs and serum lipid levels in the control group (*P* ≥ 0.008 for all).

### Interactions Between the *SYTL3*-*SLC22A3* SNPs and Several Environmental Factors on Serum Lipid Parameters and the Risk of CAD/IS

As listed in Table 5, several interactions between the *SYTL3*-*SLC22A3* SNPs and some environmental factors on serum lipid parameters were detected in the control group. The interactions of the *SYTL3* rs6455600-smoking, *SYTL3* rs6455600-BMI, and *SYTL3* rs9456350-BMI affected TG levels. The interaction of the *SYTL3* rs2129209- and *SLC22A3* rs539298-BMI influenced TC levels; and the interaction of the *SYTL3* rs9364496-smoking or *SYTL3* rs9364496-BMI influenced TG or TC levels. The interaction of the *SYTL3* rs2129209-, *SYTL3* rs9456350- and *SLC22A3* rs446809-alcohol consumption influenced HDL-C levels; and the interaction of the *SLC22A3* rs539298-alcohol consumption affected HDL-C and ApoA1 levels.

**Table 5:** The *P*-values for the interactions of genotypes and gender, age, drinking, smoking, and BMI on serum lipid levels and the risk of CAD and IS.

**SNP/factor**	**Lipid**							**CAD**	**IS**
	**TC**	**TG**	**HDL-C**	**LDL-C**	**ApoA1**	**ApoB**	**ApoA1/ApoB**		
**rs9364496**									
Gender	0.622	0.028	0.004	0.022	0.022	0.003	0.031	0.052	0.089
Age	0.793	0.153	0.645	0.478	0.478	0.275	0.444	0.550	0.430
Smoking	0.004	0.000	0.003	0.002	0.002	0.877	0.136	0.174	0.156
Drinking	0.399	0.020	0.956	0.040	0.023	0.278	0.401	0.022	0.017
BMI	0.000	0.003	0.092	0.006	0.006	0.610	0.083	0.019	0.005
**rs6455600**									
Gender	0.655	0.004	0.004	0.022	0.002	0.071	0.388	0.286	0.366
Age	0.684	0.056	0.828	0.681	0.228	0.992	0.374	0.216	0.164
Smoking	0.473	0.000	0.089	0.002	0.424	0.293	0.013	0.251	0.363
Drinking	0.359	0.030	0.042	0.003	0.056	0.906	0.374	0.221	0.265
BMI	0.006	0.001	0.304	0.010	0.144	0.101	0.004	0.010	0.131
**rs2129209**									
Gender	0.364	0.063	0.012	0.008	0.235	0.021	0.659	0.605	0.005
Age	0.900	0.428	0.688	0.497	0.198	0.479	0.266	0.004	0.407
Smoking	0.849	0.005	0.105	0.011	0.798	0.296	0.061	0.880	0.654
Drinking	0.373	0.002	0.000	0.023	0.102	0.500	0.115	0.020	0.189
BMI	0.001	0.013	0.207	0.006	0.235	0.082	0.006	0.016	0.031
**rs9456350**									
Gender	0.281	0.098	0.021	0.017	0.013	0.026	0.285	0.809	0.162
Age	0.916	0.561	0.084	0.637	0.083	0.679	0.230	0.039	0.281
Smoking	0.933	0.027	0.010	0.023	0.126	0.293	0.027	0.494	0.037
Drinking	0.108	0.010	0.000	0.020	0.017	0.412	0.047	0.008	0.030
BMI	0.011	0.001	0.032	0.006	0.106	0.072	0.005	0.130	0.071
**rs446809**									
Gender	0.314	0.090	0.009	0.005	0.004	0.011	0.013	0.890	0.759
Age	0.215	0.266	0.286	0.009	0.170	0.191	0.009	0.768	0.298
Smoking	0.294	0.032	0.054	0.007	0.463	0.018	0.024	0.281	0.381
Drinking	0.057	0.040	0.001	0.012	0.183	0.049	0.003	0.113	0.167
BMI	0.004	0.013	0.112	0.021	0.225	0.010	0.004	0.005	0.076
**rs539298**									
Gender	0.678	0.002	0.004	0.032	0.002	0.075	0.630	0.781	0.738
Age	0.385	0.131	0.052	0.839	0.033	0.928	0.343	0.006	0.180
Smoking	0.066	0.689	0.030	0.011	0.193	0.374	0.089	0.216	0.143
Drinking	0.097	0.447	0.000	0.003	0.000	0.890	0.103	0.000	0.000
BMI	0.000	0.002	0.045	0.010	0.058	0.003	0.007	0.334	0.200

*SNP, single nucleotide polymorphism; TC, total cholesterol; TG, triglyceride; HDL-C, high-density lipoprotein cholesterol; LDL-C, low-density lipoprotein cholesterol; ApoA1, apolipoprotein A1; ApoB, apolipoprotein B; CAD, coronary artery disease; IS, ischemic stroke; BMI, body mass index. P ≤ 0.00125 was considered statistically significant after Bonferroni correction*.

**Figure 5:**
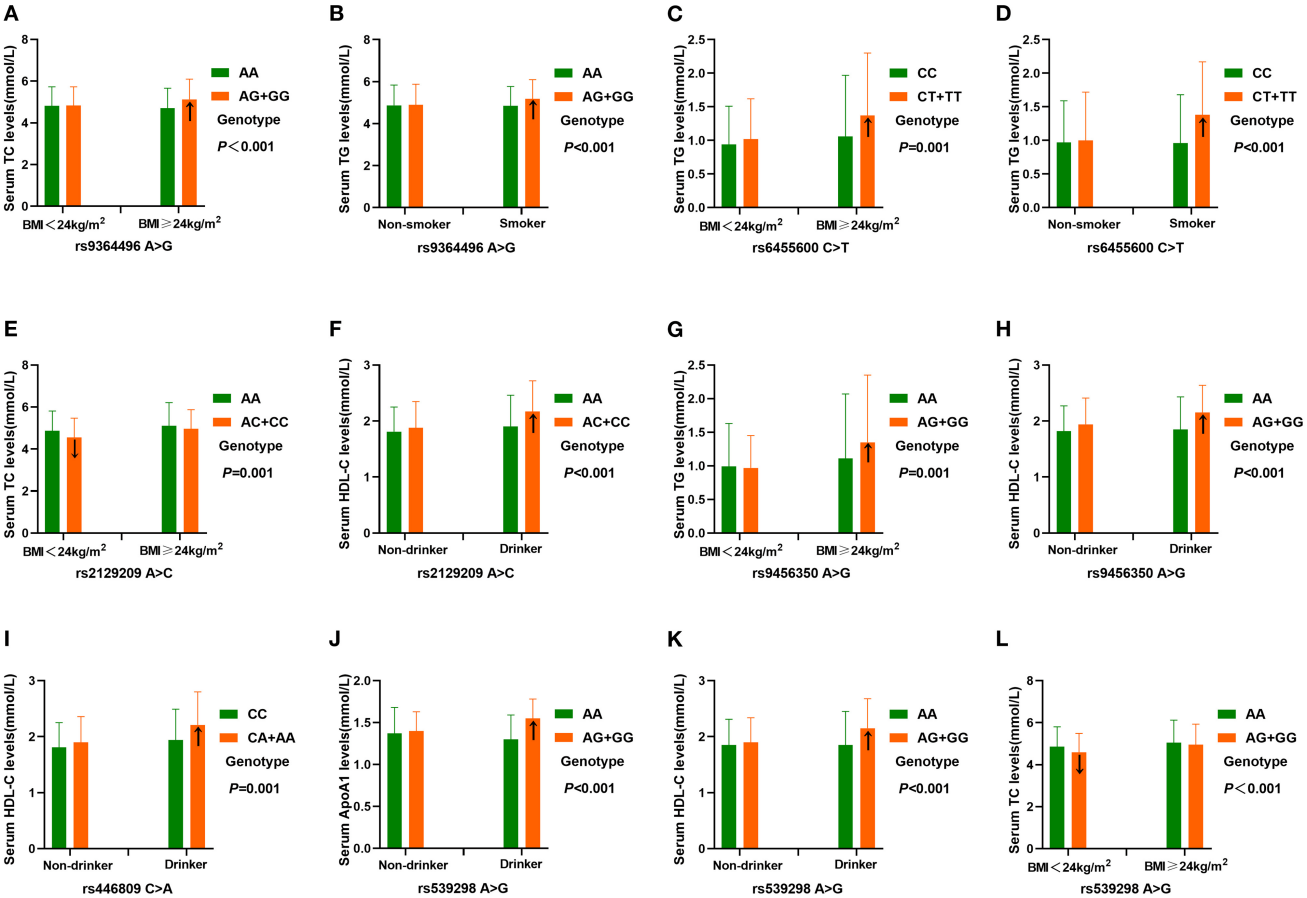
The interactions of the *SYTL3*-*SLC22A3* SNPs and drinking, BMI and smoking on serum lipid levels. TC, total cholesterol; TG, triglyceride; BMI, body mass index; ApoA1, apolipoprotein A1; HDL-C, high-density lipoprotein cholesterol. A *P* ≤ 0.00125 (corresponding to *P* < 0.05 after adjusting for five environmental exposures multiplied by eight outcomes by the Bonferroni correction) was considered statistically significant. **(A)**, rs9364496-BMI on TC; **(B)**, rs9364496-smoking on TG; **(C)**, rs6455600-BMI on TG; **(D)**, rs6455600-smoking on TG; **(E)**, rs2129209-BMI on TC; **(F)**, rs2129209-drinking on HDL-C; **(G)**, rs9456350-BMI on TG; **(H)**, rs9456350-drinking on HDL-C; **(I)**, rs446809-drinking on HDL-C; **(J)**, rs539298-drinking on ApoA1; **(K)**, rs539298-drinking on HDL-C; **(L)**, rs539298-BMI on TC.

As presented in Figure 5, the interactions of the *SYTL3* rs6455600CT/TT-BMI ≥ 24 kg/m^2^ or smoking, and *SYTL3* rs9456350AG/GG-BMI ≥ 24 kg/m^2^ increased TG levels. The interactions of the *SLC22A3* rs539298AG/GG-BMI <24 kg/m^2^, and *SYTL3* rs2129209AC/CC-BMI <24 kg/m^2^ reduced TC levels; and the interaction of *SYTL3* rs9364496AG/GG-BMI ≥ 24 kg/m^2^ or smoking increased TC or TG levels, respectively. The interactions of the *SYTL3* rs9456350AG/GG-, *SLC22A3* rs446809CA/AA-, and *SYTL3* rs2129209AC/CC-alcohol consumption increased HDL-C levels; and the interaction of the *SLC22A3* rs539298AG/GG-alcohol consumption increased HDL-C and ApoA1 levels.

### Relative Factors for Serum Lipid Parameters

**Figure 6:**
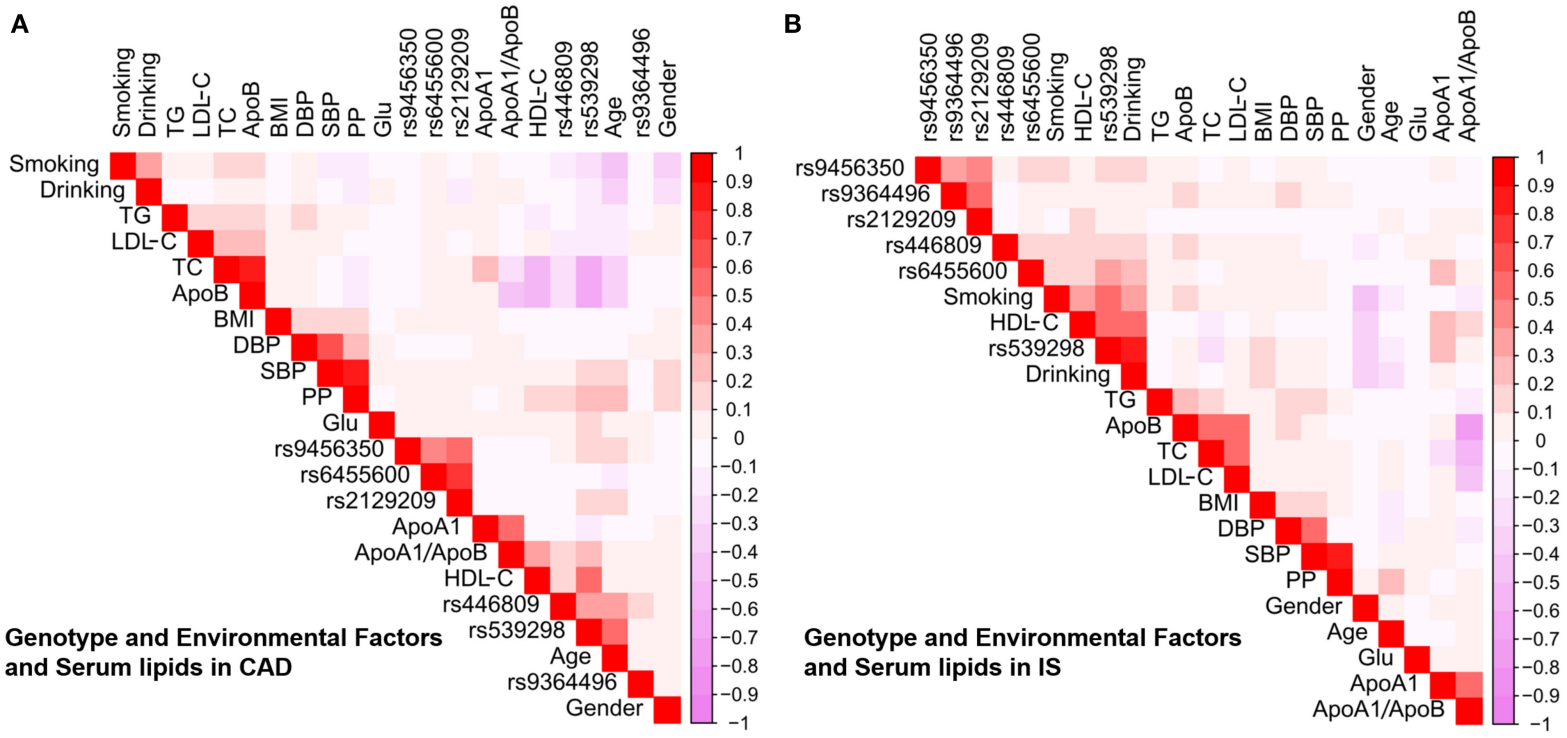
Association between environmental exposures as well as the candidate loci and serum lipid parameters in CAD **(A)** or IS **(B)**. CAD, coronary artery disease; IS, ischaemic stroke. SBP, systolic blood pressure; TC, total cholesterol; BMI, body mass index; TG, triglyceride; ApoA1/B, the ratio of apolipoprotein A1 to apolipoprotein B; HDL-C, high-density lipoprotein cholesterol; DBP, diastolic blood pressure; LDL-C, low-density lipoprotein cholesterol; ApoA1, apolipoprotein A1; PP, pulse pressure; ApoB, apolipoprotein B; Glu, glucose.

As shown in Figure 6, Pearson correlation analysis revealed that there were significant correlations between the 6 SNPs and several environmental factors, including alcohol consumption, BMI, cigarette smoking, blood glucose, age, blood pressure, sex and serum lipid profiles, in the CAD (Figure 6A) and IS groups (Figure 6B).

## Discussion

The main findings of the current research included the following aspects: (1) The allelic and genotypic frequencies of the *SYTL3* rs2129209 and *SLC22A3* rs539298 SNPs were different between controls and CAD/IS patients. The rs2129209AC/CC genotype and rs2129209C allele frequencies as well as the rs539298AG/GG genotype and rs539298G allele frequencies were lower in CAD/IS patients than in controls, respectively. (2) The *SLC22A3* rs539298 SNP was correlated with TC levels in the control group, the rs539298G allele carriers had lower TC levels than the rs539298G allele non-carriers. At the same time, the *SLC22A3* rs539298 SNP interacted with alcohol consumption would reduce the risk of CAD and IS. (3) Several haplotypes were associated with an increased or decreased risk of CAD and IS, and the haplotypes could explain more changes in the risk of CAD/IS than any single SNP alone. In addition, the interactions between several haplotypes and different environmental factors on the onset of CAD and IS were also observed. (4) The interactions between the *SYTL3*-*SLC22A3* SNPs and several environmental factors, including smoking, alcohol consumption and BMI, could affect serum lipid parameters such as TC, TG, HDL-C and ApoA1 levels.

The genotypic and allelic frequencies of the *SLC22A3* rs539298 SNP in different racial/ethnic groups are not well-known. Based on the data derived from the International 1000 Genomes database (https://www.ncbi.nlm.nih.gov/variation/tools/1000genomes/), the frequencies of the rs539298G allele and the AG, GG genotypes were, respectively, 52.21, 49.56, and 27.43% in Europeans; 30.23, 41.86 and 9.30% in Japanese; 59.82, 53.57, and 33.04% in Sub-Saharan Africans; 52.04, 59.18, and 22.45% in Americans of African Ancestry in the Southwestern USA (ASW); 39.20, 48.86, and 14.77% in Gujarati Indians in Houston (GIH); 51.67, 45.56, and 28.89% in Luhya in Webuye, Kenya (LWK); 49.00, 46.00, and 25.00% in those with Mexican ancestry in Los Angeles, California (MEX); 58.10, 50.00, and 33.10% in Maasai in Kinyawa, Kenya (MKK); and 26.83, 48.84, and 4.65% in Han Chinese in Beijing (CHB). In the current study, we noticed that the allele and genotype frequencies of the *SLC22A3* rs539298 SNP were significantly different between the controls and cases. The frequencies of the G allele and AG and GG genotypes in our study populations were 28.28, 41.99, and 7.28% in controls; 23.68, 36.81, and 5.28% in CAD patients; and 22.69, 35.05, and 5.16% in IS cases (*P* < 0.05–0.001, respectively). These findings suggest that the *SLC22A3* rs539298 SNP may have racial/ethnic and population specificity. However, these findings need to be confirmed in other populations or ethnic groups with larger sample size.

Hyperlipidaemia is closely related to the occurrence and development of atherosclerotic cardiovascular and cerebrovascular diseases (39, 40). Previous studies showed that the *SLC22A3* rs2048327 SNP was significantly associated with cardiovascular disease events in familial hypercholesterolemia subjects (41). A genome-wide haplotype association (GWHA) research revealed that the *SLC22A3*-*LPAL2*-*LPA* cluster was a strong susceptibility locus for CAD (42). Furthermore, Wang, et al. suggested that the rs3088442G allele might inhibit miR-147a binding to the 3' UTR region of *SLC22A3*, resulting in increased the expression levels of *SLC22A3*, and ultimately lead to increased risk of CAD (43). In addition, another case control study noticed that four SNPs (rs2048327, rs3127599, rs7767084 and rs10755578) in the *SLC22A3*-*LPAL2*-*LPA* cluster were not significantly associated with the risk of CAD (44). However, no study has yet well-elucidated the potential association between the six selected SNPs in the *SYTL3*-*SLC22A3* cluster and serum lipid levels and the risk of CAD and IS. To meet this need, the potential correlation between the six selected SNPs in the *SYTL3*-*SLC22A3* cluster and serum lipid levels in humans has been well-elaborated in our previous research (45). We noticed that the rs6455600, rs2129209 and rs539298 SNPs were associated with TC levels; and the rs446809 SNP was associated with TG and LDL-C levels in the Chinese Han population. Furthermore, we also observed that the rs539298G allele carriers had lower serum TC levels than the rs539298G allele non-carriers, and the dominant model of the rs539298 SNP reduced the morbidity of hyperlipidaemia in the Chinese Han population. In the current research, we obtained the similar findings to our previous study. We noticed that serum TC levels in the control group were significantly different between the rs539298AA and rs539298AG/GG genotypes, and participants with the rs539298AG/GG genotypes had lower serum TC levels than those with the rs539298AA genotype. In addition, we also found that the rs539298G allele carriers had lower risk of CAD (adjusted OR = 0.74, 95% CI: 0.60–0.91) and IS (adjusted OR = 0.67, 95% CI: 0.54–0.83) than the rs539298G allele non-carriers. These findings suggested that the *SLC22A3* rs539298A allele may be a genetic risk factor for ischaemic cardiovascular and cerebrovascular diseases, and the *SLC22A3* rs539298G allele may reduce the risk of CAD and IS by affecting serum TC levels. However, the correlation between the *SLC22A3* rs539298 SNP and serum TC levels needs to be confirmed by proteomics studies in future research.

Haplotype analysis showed that the *SYTL3*-*SLC22A3* A-C-A-A-A-A, G-T-C-G-C-A and A-T-A-A-C-A haplotypes increased the risk of CAD, while the *SYTL3*-*SLC22A3* A-C-A-A-C-G haplotype reduced the risk of CAD. Meanwhile, the *SYTL3*-*SLC22A3* A-C-A-A-A-A, G-T-C-G-A-G and A-T-A-A-C-A haplotypes increased the risk of IS, whereas the *SYTL3*-*SLC22A3* A-C-A-A-A-G and A-C-A-A-C-G haplotypes reduced the risk of IS. These results suggest that haplotypes could explain more changes in the risk of CAD/IS than any single SNP alone.

A large number of studies have suggested that as a multifactorial and complex disorder, CAD or IS occurs due to numerous pathogenic factors, including genetic background, environmental exposures and their interactions (46–48). However, the potential mutual effect between the *SLC22A3* rs539298 SNP and environmental factors on blood lipid levels and the risk of CAD and IS is still unknown. In the current research, we firstly noticed that the interaction of the *SLC22A3* rs539298-alcohol consumption increased serum ApoA1 as well as HDL-C levels; and *SLC22A3* rs539298-BMI <24 kg/m^2^ reduced serum TC levels. These findings could partly account for a decreased risk of CAD and IS in rs539298G allele carriers. Several other potential interactions between haplotypes and environmental factors on the risk of CAD and IS were also observed in this study. The interactions between the *SYTL3*-*SLC22A3* A-C-A-A-A-A and A-T-A-A-C-A haplotypes and age > 75 and smoking increased the risk of CAD. The interactions between the *SYTL3*-*SLC22A3* G-T-C-G-C-A haplotype and smoking and BMI ≥ 24 kg/m^2^ increased the risk of CAD. The interaction between the *SYTL3*-*SLC22A3* A-C-A-A-C-G haplotype and alcohol consumption reduced the risk of CAD. In addition, the interaction between the *SYTL3*-*SLC22A3* A-C-A-A-A-G haplotype and female sex and alcohol consumption reduced the risk of IS. The interactions between the *SYTL3*-*SLC22A3* A-C-A-A-A-A and smoking and hypertension increased the risk of IS. The interactions between the *SYTL3*-*SLC22A3* A-T-A-A-C-A and smoking and age > 75 increased the risk of IS. The interactions between the *SYTL3*-*SLC22A3* A-C-A-A-C-G haplotype and hypertension increased the risk of IS, while its interaction with alcohol consumption reduced the risk of IS. However, these findings have yet to be confirmed by additional research.

The current study may have several limitations. First, compared to other studies, the number of controls and patients was relatively small. Larger samples are necessary to validate our results in future research. Second, most of the patients with CAD or IS received some secondary prevention drugs. All of these drugs may have a certain effect on blood lipid levels. Third, to improve the accuracy of the statistical analysis, we adjusted for several environmental factors, such as BMI, age, smoking, sex and alcohol consumption, but the potential effects of the above factors on blood lipid levels and the risk of CAD and IS could not be completely eliminated. Finally, *in vitro* and *in vivo* studies are necessary to strengthen the significance of our results.

## Conclusions

In conclusion, the current research showed that the allelic and genotypic frequencies of the *SLC22A3* rs539298 SNP were significantly different between controls and CAD/IS patients. The *SLC22A3* rs539298G carriers in the control group had lower serum TC levels than the G allele non-carriers. The interaction between the *SLC22A3* rs539298 SNP and alcohol consumption reduced the risk of CAD and IS. In addition, haplotypes could explain more changes in the risk of CAD/IS than any single SNP alone. Several potential interactions between the haplotypes and some environmental factors affected the risk of CAD and IS. These findings revealed that the *SLC22A3* rs539298A allele may be a new genetic marker for CAD and IS in our study populations. The correlation between the *SLC22A3* rs539298 SNP and CAD/IS may be partly explained by its association with decreased serum TC levels in this study.

## Data Availability Statement

The datasets presented in this study can be found in online repositories. The names of the repository/repositories and accession number(s) can be found in the article/Supplementary Material.

## Ethics Statement

The studies involving human participants were reviewed and approved by Ethics Committee of the First Affiliated hospital, Guangxi Medical University. The patients/participants provided their written informed consent to participate in this study.

## Author Contributions

P-FZ conceived the study, participated in the design, performed the statistical analyses, and drafted the manuscript. R-XY conceived the study, participated in the design, carried out the epidemiological survey, collected the samples, and helped to draft the manuscript. X-LC, W-XC, J-ZW, and FH carried out the epidemiological survey and collected the samples. All authors read and approved the final manuscript.

## Conflict of Interest

The authors declare that the research was conducted in the absence of any commercial or financial relationships that could be construed as a potential conflict of interest.

## Publisher's Note

All claims expressed in this article are solely those of the authors and do not necessarily represent those of their affiliated organizations, or those of the publisher, the editors and the reviewers. Any product that may be evaluated in this article, or claim that may be made by its manufacturer, is not guaranteed or endorsed by the publisher.

## Abbreviations

ANCOVA: covariance analysis
Apo: apolipoprotein
BMI: body mass index
CAD: coronary artery disease
CI: confidence interval
DNA: deoxyribonucleic acid
GWAS: genome-wide association study
HDL-C: high-density lipoprotein cholesterol
LDL-C: low-density lipoprotein cholesterol
OR: odds ratio
*SYTL3*: synaptotagmin like 3
*SLC22A3*: solute carrier family 22 member 3
SNP: single nucleotide polymorphisms
T2DM: type 2 diabetes mellitus
TC: total cholesterol
TG: triglyceride
AMI: acute myocardial infarction
MRI: magnetic resonance imaging
MI: myocardial infarction
LD: linkage disequilibrium
NGS: next-generation sequencing
ASW: Americans of African Ancestry in the Southwestern USA
GIH: Gujarati Indians in Houston
LWK: Luhya in Webuye, Kenya
MEX: Mexican ancestry in Los Angeles, California
MEX: Maasai in Kinyawa, Kenya
CHB: Han Chinese in Beijing.
