# Supplementary material for: Effect of SYTL3-SLC22A3 Variants, Their Haplotypes, and G × E Interactions on Serum Lipid Levels and the Risk of Coronary Artery Disease and Ischaemic Stroke
Source: Front Cardiovasc Med. 2021 Aug 12;8:713068. doi: 10.3389/fcvm.2021.713068 (PMC8387813; doi:10.3389/fcvm.2021.713068)
Supplement: Supplementary file 1 [file Table_1.DOCX]

**Multiplex PCR and sequencing**

A panel which contains 6 target SNP sites was designed. Library preparation was performed by two step PCR. First round PCR reaction was set up as follows: DNA (10 ng/μl) 2 μl; amplicon PCR forward primer mix (10 μM) 1μl; amplicon PCR reverse primer mix (10 μM) 1 μl; 2 × PCR Ready Mix 15 μl (total 25 μl; Kapa HiFi Ready Mix). The plate was sealed and PCR performed in a thermal instrument (BIO-RAD, T100TM) using the following program: 1 cycle of denaturing at 98 °C for 5 min, first 8 cycles of denaturing at 98 °C for 30 s, annealing at 50 °C for 30 s, elongation at 72 °C for 30 s, then 25 cycles of denaturing at 98 °C for 30 s, annealing at 66 °C for 30 s, elongation at 72 °C for 30 s and a final extension at 72 °C for 5 min. Finally hold at 4℃. The PCR products were checked using electrophoresis in 1 % (w/v) agarose gels in TBE buffer (Tris, boric acid, EDTA) stained with ethidium bromide (EB) and visualized under UV light. Then we used AMPure XP beads to purify the amplicon product. After that, the second round PCR was performed. PCR reaction was set up as follows: DNA (10 ng/μl) 2 μl; universal P7 primer with barcode (10 μM) 1μl; universal P5 primer (10 μM) 1μl; 2 × PCR Ready Mix 15 μl (total 30 μl; Kapa HiFi Ready Mix). The plate was sealed and PCR performed in a thermal instrument (BIO-RAD, T100TM) using the following program: 1 cycle of denaturing at 95 °C for 3 min, then 5 cycles of denaturing at 94 °C for 30 s, annealing at 55 °C for 20 s, elongation at 72 °C for 30 s , elongation at 72 °C for 30 s and a final extension at 72 °C for 5 min. Then we used AMPure XP beads to purify the amplicon product. The libraries were then quantified and pooled. Paired-end sequencing of the library was performed on the HiSeq XTen sequencers (Illumina, San Diego, CA).

**Supplementary Table 1.** The PCR primers for Next-Generation Sequencing

| **SNP** | **Forward primer** | **Reverse primer** |
| --- | --- | --- |
| ***SYTL3* rs9364496** | CCTGGCCTGTTGTATTTATAAGCA | CTCCTCCATGACCCTTCTAAGAGT |
| ***SYTL3* rs6455600** | GTACCATATTCTCTGGAGGTTTTAGACAC | TGGATTGATAATGAATCTGCTGACACA |
| ***SYTL3* rs2129209** | GCCTCAATAAATGTTTGTTGAGTGATT | CGCCTGTGGGATAGTGTGAAAAG |
| ***SYTL3* rs9456350** | TTTTCTTGTGTCCTCCCATGGTAG | CCTGCGTGGAAAGCCAAATTTTT |
| ***SLC22A3* rs446809** | AGGCATTAATCTCAAGGACACTCTT | GTGAAGCCATTAGACTTCACTTCTTAGT |
| ***SLC22A3* rs539298** | ACTTTCTTCGAAGCCGAGTTGTAA | CCCTTGCATTTTGAGTAACCATTTGG |

*SYTL3*, synaptotagmin like 3; *SLC22A3,* solute carrier family 22 member 3.
